# Using Machine Learning to Identify the Dynamic Evolution Patterns of Negative Emotions in Perinatal Women: A Longitudinal Study in Southwest China

**DOI:** 10.1002/mco2.70331

**Published:** 2025-08-15

**Authors:** Yuan Zhang, Wenlong Li, Jian Zou, Guohui Yang, Xiaoni Zhong, Biao Xie

**Affiliations:** ^1^ Department of Epidemiology and Health Statistics School of Public Health, Chongqing Medical University Chongqing China; ^2^ Research Center for Medicine and Social Development Chongqing Medical University Chongqing China

**Keywords:** longitudinal trajectory, machine learning, negative emotions, perinatal women, prediction

## Abstract

Paying attention to the mental health of perinatal women is helpful in improving their quality of life. However, the existing research pays less attention to the heterogeneity of its negative emotional trajectory and the identification of high‐risk groups. This study recruited 860 perinatal women from four large hospitals in Chongqing from March 2018 to January 2019. They were followed up by structured questionnaires in the first trimester, second trimester, third trimester, and about 6 weeks after delivery. The growth mixture model was used to analyze the developmental trajectory of negative emotions, and six machine learning algorithms were used to establish a high‐risk negative emotion recognition model. The performance of the model was comprehensively evaluated by five performance indicators. The SHAP algorithm was used to explain the model. Negative emotional trajectories were divided into four categories: low‐stable anxiety group, gradually increasing high‐anxiety group, mild sustained depression group, and high‐progressive depression group. The extreme gradient boosting model performed best, with the highest prediction performance score (24 points). In summary, the negative emotional trajectory of perinatal women is dynamic and heterogeneous, and the prediction model based on machine learning may play an important role in identifying high‐risk negative emotions.

## Graphical abstract

This study examined the dynamic evolution of negative emotions in perinatal women. By utilizing a growth mixture model on a longitudinal cohort of pregnant women in Southwest China, we accurately identified four distinct trajectories of negative emotional development. Additionally, we explored the potential of machine learning models to predict high‐risk negative emotions.

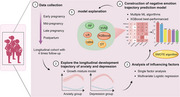

## 1 Introduction

Negative emotions are fundamental subjective experiences characterized by feelings of sadness and distress, encompassing a range of unpleasant emotional states such as anger, fear, anxiety, and depression. Among these, anxiety and depression are prevalent emotional disorders in the perinatal period [1, 2], and often co‐occur [3]. Meta‐analysis showed that the incidence of anxiety in pregnant women gradually increased from 18.2% in the first trimester to 19.1% in the second trimester and 24.6% in the third trimester [4]. The incidence of perinatal anxiety disorders is 27.6% in low‐and middle‐income countries, 24.0% in low‐income countries, and 19.1% in upper‐middle‐income countries [5]. Additionally, epidemiological surveys indicated that the global incidence of postpartum depression ranges from 5.0% to 26.32% [6], with a prevalence rate of 14.8% reported in China [7]. Recent statistics revealed that 32.58% of pregnant women in China experience prenatal depression, while 12.9% suffer from early anxiety disorders. Furthermore, the likelihood of complications associated with postpartum depression can be as high as 50% to 80% [8]. This data underscores the significant psychological challenges faced by pregnant women in China.

It is important to note that symptoms of depression and anxiety in pregnant women, even at subclinical levels, can significantly impact the early development of infants. These effects may include abnormal crying behavior, sleep disorders, and feeding difficulties [9]. The adverse psychological state of elderly pregnant women can increase the risk of negative maternal and infant outcomes, such as gestational hypertension, gestational diabetes, cesarean delivery, premature rupture of membranes, postpartum hemorrhage, fetal distress, and low birth weight [10, 11, 12]. Therefore, it is crucial to assess symptoms of anxiety and depression early in pregnancy to better understand their progression over time and to prevent potential postpartum complications in Chinese pregnant women.

Although existing studies have focused on the heterogeneity of negative emotions in perinatal women, most mental health research still relies on cross‐sectional designs. Additionally, some studies are limited by convenience sampling and short follow‐up periods [13, 14]. Only a few investigations have reported the developmental trajectories of women at different stages of pregnancy. For instance, McCall‐Hossenfeld et al. [15] found that marital status was associated with the severity of depressive symptom trajectories. For every unit increase in pregnancy stress, the likelihood of pregnant women entering the high‐anxiety group or the high‐depression group increased by 1.09 times and 1.13 times, respectively [16, 17]. Furthermore, low social support, inadequate family care, and a rural living environment are significant predictors of high anxiety and high depression trajectories [18]. The study by Yang et al. [19] demonstrated that a low level of psychological resilience also increases the likelihood of perinatal women entering the high‐level depressive symptom trajectory group. The heterogeneity of negative emotional trajectories not only reflects the dynamic changes in symptoms but also is closely related to the quality of life. Research has shown that anxiety and depression are significantly associated with higher healthcare costs [20, 21], an increased risk of physical complications (such as cardiovascular disease), and a decreased quality of life [22, 23, 24, 25]. Therefore, an in‐depth study of the developmental trajectories and predictive factors of negative emotions is crucial for identifying high‐risk groups, implementing targeted interventions, and improving the quality of life for perinatal women [26, 27].

**FIGURE 1:**
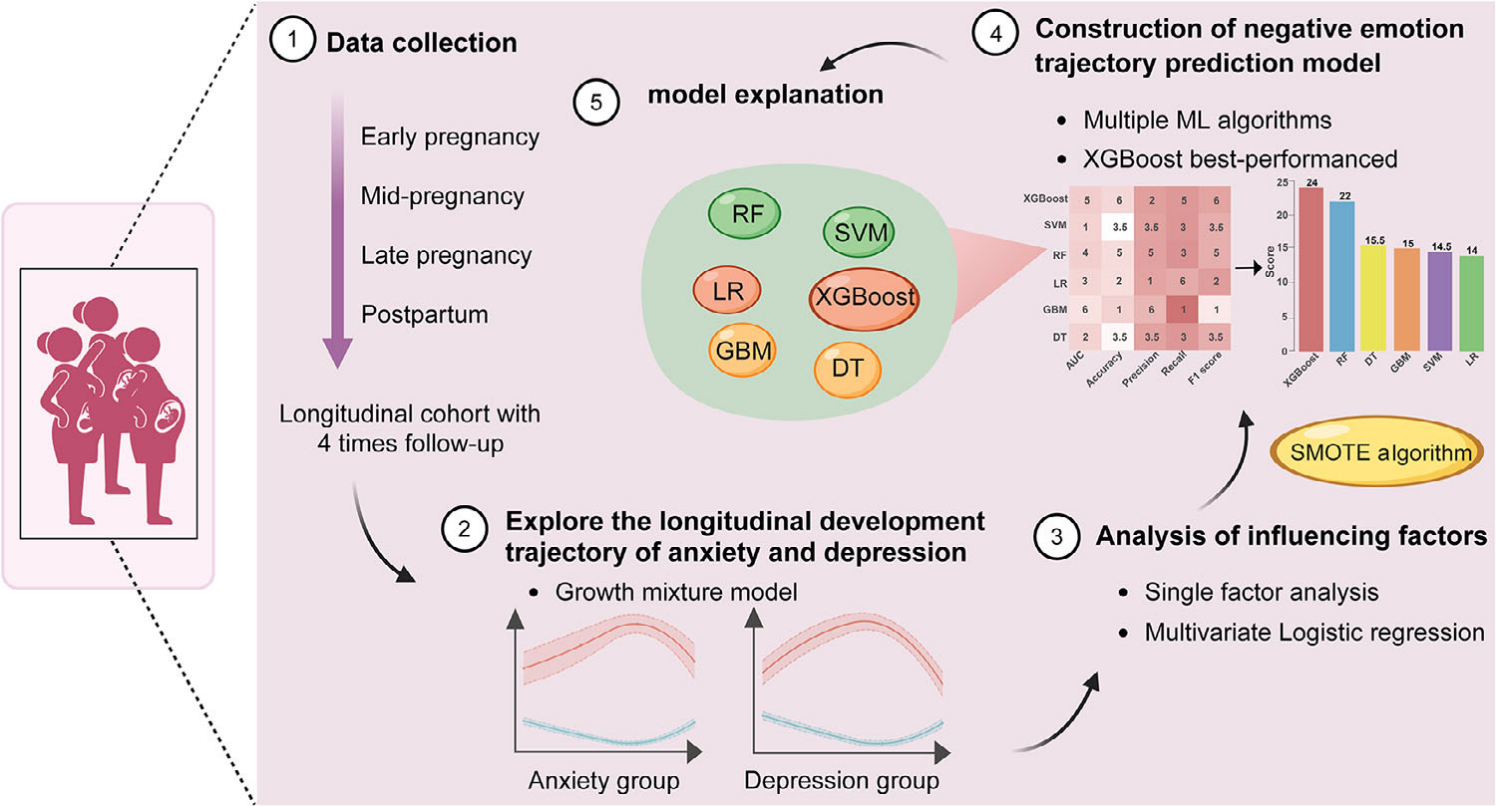
The design flow chart of this study. Image created with BioRender.com, with permission. ML, machine learning; RF, random forest; SVM, support vector machine; DT, decision tree; LR, logistic regression; XGBoost, extreme gradient boosting; GBM, gradient boosting machine; SMOTE, synthetic minority oversampling technique.

In this study, we conducted a multicenter longitudinal study among perinatal women in Chongqing, China, to explore the dynamic developmental trajectories of various negative emotions experienced by these women. We also analyzed the influence of multiple influencing factors on these different trajectories. Based on our findings, we employed a range of machine learning (ML) algorithms to develop a high‐risk identification model aimed at accurately identifying the high‐risk groups for negative emotions among perinatal women. This model serves as a foundation for the development of effective population intervention measures and comprehensive prevention and control measures. The research flow chart is shown in Figure 1.

## 2 Results

### 2.1 Baseline Characteristics

This study excluded samples with missing visits in any period and with an overall missingness rate of more than 20%, and after interpolating data with less than 20% missingness, a total of 860 longitudinal data points with complete information were eventually obtained. The basic characteristics of the subjects are shown in Table 1. Women under 30 years old accounted for the main part (85.93%), most of them were from the Han nationality (97.67%), and only 20 women were minorities. Among them, 75.47% of women's body mass index (BMI) was between 18.5 and 25, 16.28% of women were underweight, and 8.25% of women were overweight. In terms of the distribution of residence, urban women account for 61.86%, while rural women account for 38.14%. In terms of education level, 61.86% of women have a high school/technical secondary school or below, and 38.14% of women have received higher education. About half (51.63%) of women are currently out of work. About 41.51% of women's family monthly income is 3001–5000 yuan, 32.56% of women's family monthly income is more than 5000 yuan, and 25.93% of women's family monthly income is less than 3000 yuan. 60.12% used medical insurance as a medical payment method. Most women had no drinking history (98.84%), no active smoking history (96.98%), and no previous medical history (93.26%). First‐time pregnant women accounted for 28.14% of the total, and more than two‐thirds of women had a history of pregnancy (71.86%).

**TABLE 1:** Basic characteristics of research objects.

	Classification	Quantity	Proportion (%)
Ethnic groups	Han nationality	840	97.67
	Other nationalities	20	2.33
Age	≤25	373	43.37
	25–30	366	42.56
	≥30	121	14.07
BMI	<18.5	140	16.28
	18.5–25	649	75.47
	>25	71	8.25
Residence	Town	532	61.86
	Rural	328	38.14
Educational level	Junior high school and below	278	32.33
	High school/vocational high school/technical secondary school	254	29.53
	Junior college/undergraduate and above	328	38.14
Profession	On the job	416	48.37
	Housewives/unemployed	444	51.63
Family per capita monthly income	≤3000	223	25.93
	3001–5000	357	41.51
	≥5001	280	32.56
Medical expenses payment method	At his own expense	343	39.88
	Medical insurance	517	60.12
history of drinking	Yes	10	1.16
	No	850	98.84
Smoking history	Active	26	3.02
	Passive	834	96.98
Previous medical history	Yes	58	6.74
	No	802	93.26
History of pregnancy	Yes	618	71.86
	No	242	28.14

The incidence of anxiety and depression in women during the perinatal period is shown in Table S1. The perinatal period is a stage of women's physical and psychological challenges, especially mental health problems such as anxiety and depression. The data showed that the positive rate of anxiety symptoms fluctuated in different degrees in early pregnancy, middle pregnancy, late pregnancy, and postpartum, which were 13.95%, 11.63%, 10.12% and 11.63%, respectively. At the same time, the positive rate of depressive symptoms also showed a similar trend: 5.12%, 4.53%, 3.37%, and 8.84%, respectively. These data reveal the dynamic changes in pregnant women's mental health at different stages of pregnancy  .

### 2.2 Identification of Anxiety Developmental Trajectory

**FIGURE 2:**
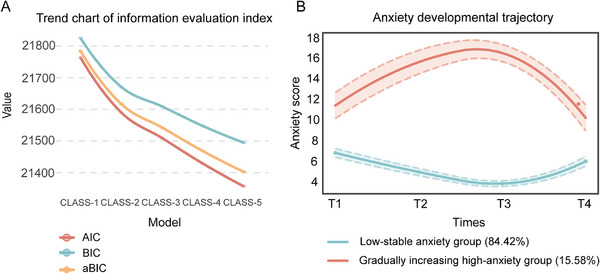
CLASS‐1‐CLASS‐5 anxiety group trajectory changes. (A) The line chart shows the change in CLASS‐1–CLASS‐5 evaluation index. (B) The results of CLASS‐1 and CLASS‐2 trajectory analysis in perinatal women. The horizontal coordinate was the follow‐up time, and the vertical coordinate was the anxiety symptom score. The red line represents “CLASS‐1” (n=726, 84.42%). The blue line represents “CLASS‐2” (*n* = 134, 15.58%). AIC, Akaike Information Criterion; BIC, Bayesian Information Criterion; aBIC: adjusted Bayesian Information Criterion.

To better understand the changes of anxiety symptoms of pregnant women over time, this study used the total anxiety scores of four measurements to divide the population into one category (CLASS‐1) by growth mixture model (GMM), and increased the number of categories in turn to form a model of 2 categories (CLASS‐2), 3 categories (CLASS‐3), 4 categories (CLASS‐4) and 5 categories (CLASS‐5). The following evaluation index system is used for model selection: Akaike information criterion (AIC), Bayesian information criterion (BIC), and sample‐size adjusted BIC (aBIC) are classified as evaluation index system 1; Entropy, Lo‐Mendell‐Rubin (LMR) and bootstrapped likelihood ratio test (BLRT) are classified as evaluation index system 2. Combined with the trend change diagram of Figure 2A, the evaluation index system 1 decreases with the increase of the number of categories, and there are obvious inflection points in the three categories. At this time, the Entropy value is greater than 0.8, LMR and BLRT are statistically significant (*p *< 0.05). At the same time, Table S2 summarizes the fitting information of each category of the GMM model evaluation index system 2. The evaluation index system 2 showed good statistical significance in the CLASS‐2 model and CLASS‐3 model, calculated by the three methods (except for the three categories of quadratic estimation). However, researchers generally believe that any group in the classification results needs to contain at least 5% of the total number of samples, and the model classification results are reliable. In the CLASS‐2 model, the number of classified people estimated by the three methods is less than 5% of the number of samples, so the model is not reliable. In contrast, the CLASS‐2 model satisfies this condition. Further analysis showed that the CLASS‐2 model had the largest entropy value (entropy >0.8) in the quadratic estimation, and the LMR and BLRT values reached a significant level (*p *< 0.05), and the category probability classification was more reasonable. Therefore, we chose CLASS‐2 as the optimal model. Figure 2B showed the GMM trajectory trend of each group in the optimization model. After dividing the population trajectory into two groups by GMM, the first subgroup accounted for 84.42% of the total population. The anxiety symptoms of this group of people generally showed a trend of “initial anxiety symptoms decreased slowly, but relatively stable”, which can be named as “low‐stable anxiety group”. The second subgroup accounted for 15.6% of the total population. This part of the population had a higher level of anxiety symptoms in the early stage, and then the level of anxiety symptoms increased slowly over time, which could be named as “gradually increasing high‐anxiety group”.

### 2.3 Identification of Depression Developmental Trajectory

**FIGURE 3:**
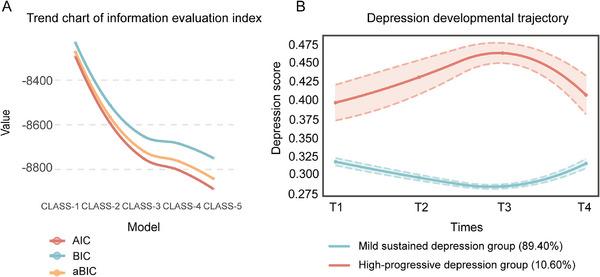
Changes in the trajectory of the depression group of CLASS‐1‐CLASS‐5. (A) The line chart shows the change in CLASS‐1–CLASS‐5 evaluation index. (B) The results of CLASS‐1 and CLASS‐2 trajectory analysis in perinatal women. The horizontal coordinate was the follow‐up time, and the vertical coordinate was the depression symptom score. The red line represented “CLASS‐1” (*n* = 769, 89.40%). The blue line represents “CLASS‐2” (*n* = 91, 10.60%).

To identify the developmental trajectory of depression, this study also used GMM to analyze the scores of each item of the scale. First, the population is divided into a class (CLASS‐1), and the evaluation index of the model is calculated. Subsequently, the number of categories was increased in turn, and the population was divided into 2 categories (CLASS‐2), 3 categories (CLASS‐3), 4 categories (CLASS‐4), and 5 categories (CLASS‐5). In the evaluation index system 1, AIC, BIC, and aBIC decreased with the increase of the number of categories, and there were obvious inflection points at the three categories (Figure 3A). From the 2‐level classification to the 3‐level classification, the index decline rate slows down, and the entropy values estimated by the three methods are all greater than 0.8. However, LMR did not reach statistical significance at all three classifications (*p *> 0.05), indicating that further increase in the number of categories may not significantly improve the model fit. Therefore, we first consider the CLASS‐2 model (Table S3). Considering the model fitting index and statistical significance test results, the CLASS‐2 model performs best in all aspects: the entropy values estimated by the three methods are all greater than 0.8. At the same time, when LMR and BLRT reached the most significant level (*p *< 0.05), the quadratically estimated CLASS‐2 model had the highest entropy value (0.943). Therefore, the CLASS‐2 model was selected as the optimal model.

The average score of the CLASS‐2 model on 10 items was further analyzed. After GMM was used to divide the population trajectory into two groups, the first subgroup accounted for 89.40% of the total population (Figure 3B). The depression level of this part of the population generally showed a trend of “depression symptoms are generally stable and mild”, which can be named as “mild sustained depression group”. The second subgroup accounted for 10.60% of the total population. The level of depressive symptoms was higher in the early stage and gradually increased over time. It can be named as the “high‐progressive depression group”. The developmental trajectories of the two subgroups are clear, indicating that the model has a good distinguishing effect on the population.

### 2.4 Predictors of Trajectory Subgroup Members

Taking the latent category of the dynamic developmental trajectory of perinatal anxiety and depression as the dependent variable, we included five dimensions: basic demographic characteristics, personal lifestyle, family factors, and social factors in the single‐factor analysis to assess their impact on the latent category of perinatal negative emotional developmental trends. Tables S4 and S5 present the results of the single‐factor analysis for anxiety and depression trajectory groupings. The influencing factors for the classification of perinatal anxiety developmental trajectory subgroups among pregnant women included: receiving one to five times of pregnant women school education, no previous medical history, moderate pregnancy stress, and moderate social support (*p *< 0.05). The OR values were 2.04 (1.20–3.45), 0.47 (0.25–0.89), 3.29 (1.27–8.52), and 0.48 (0.32–0.74), respectively.

The variables that had a significant impact on the trajectory of perinatal anxiety were: occupation, number of pregnant women, school education, past medical history, pregnancy stress, and social support (*p *< 0.05, Table 2 ).

**TABLE 2:** Multivariate logistic analysis of anxiety trajectory group.

Variables	Β	SE	Z	*p*	OR (95%CI)
Number of school education for pregnant women					
0					1.00 (Reference)
1–5	0.71	0.27	2.64	0.008^**^	2.04 (1.20–3.45)
≥6	0.43	0.66	0.65	0.51	1.54 (0.42–5.61)
Past medical history					
Yes					1.00 (Reference)
No	−0.76	0.32	−2.34	0.02^*^	0.47 (0.25–0.89)
Pregnancy pressure					
No pressure					1.00 (Reference)
Mild pressure	0.31	0.45	0.68	0.50	1.36 (0.56–3.30)
Moderate pressure	1.19	0.49	2.45	0.01^*^	3.29 (1.27–8.52)
Severe pressure	0.45	1.19	0.37	0.71	1.56 (0.15–16.22)
Social support					
Low level					1.00 (Reference)
Medium level	−0.73	0.22	−3.38	<0.001^***^	0.48 (0.32–0.74)
High level	−1.09	0.36	−3.05	0.002^**^	0.34 (0.17–0.68)

*Note*: The variables that significantly impacted the trajectory of perinatal depression included occupation, number of pregnant women's school education, degree of care of doctors and nurses, smoking history, past medical history, family care, pregnancy stress, social support, number of prenatal examinations, and domestic pets (*p* < 0.05). Additionally, the influencing factors of subgroup classification of the perinatal depression developmental trajectory were: receiving one to five times of pregnant women school education, general care of doctors and nurses, no active smoking history, no past medical history, medium level of social support and high level of social support (*p* < 0.05), The OR values for these factors were 2.30 (1.27–4.15), 2.54 (1.22–5.29), 0.32 (0.12–0.82), 0.46 (0.22–0.95), 0.38 (0.24–0.61), and 0.09 (0.03–0.30) (Table 3).

Abbreviations: OR: odds ratio, CI: confidence interval.

* *p* < 0.05.

** *p* < 0.01.

*** *p* < 0.001.

**TABLE 3:** Multivariate logistic analysis of depression trajectory group.

Variables	β	S.E	Z	*p*	OR (95%CI)
Number of school education for pregnant women					
0					1.00 (Reference)
1–5	0.83	0.30	2.76	0.006^**^	2.30 (1.27–4.15)
≥6	−14.33	570.19	−0.03	0.98	0.000 (0.000–Inf)
Degree of care of doctors and nurses					
Good					1.00 (Reference)
General	0.93	0.37	2.50	0.01^*^	2.54 (1.22–5.29)
Smoking history					
Active					1.00 (Reference)
Passive	−1.15	0.49	−2.36	0.02^*^	0.32 (0.12–0.82)
Previous medical history					
Yes					1.00 (Reference)
No	−0.78	0.37	−2.11	0.04^*^	0.46 (0.22–0.95)
Social support					
Low level					1.00 (Reference)
Medium level	−0.97	0.24	−3.98	<0.001^***^	0.38 (0.24–0.61)
High level	−2.42	0.62	−3.94	<0.001^***^	0.09 (0.03–0.30)

* *p *< 0.05.

** *p *< 0.01.

*** *p *< 0.001.

### 2.5 Model Development

**FIGURE 4:**
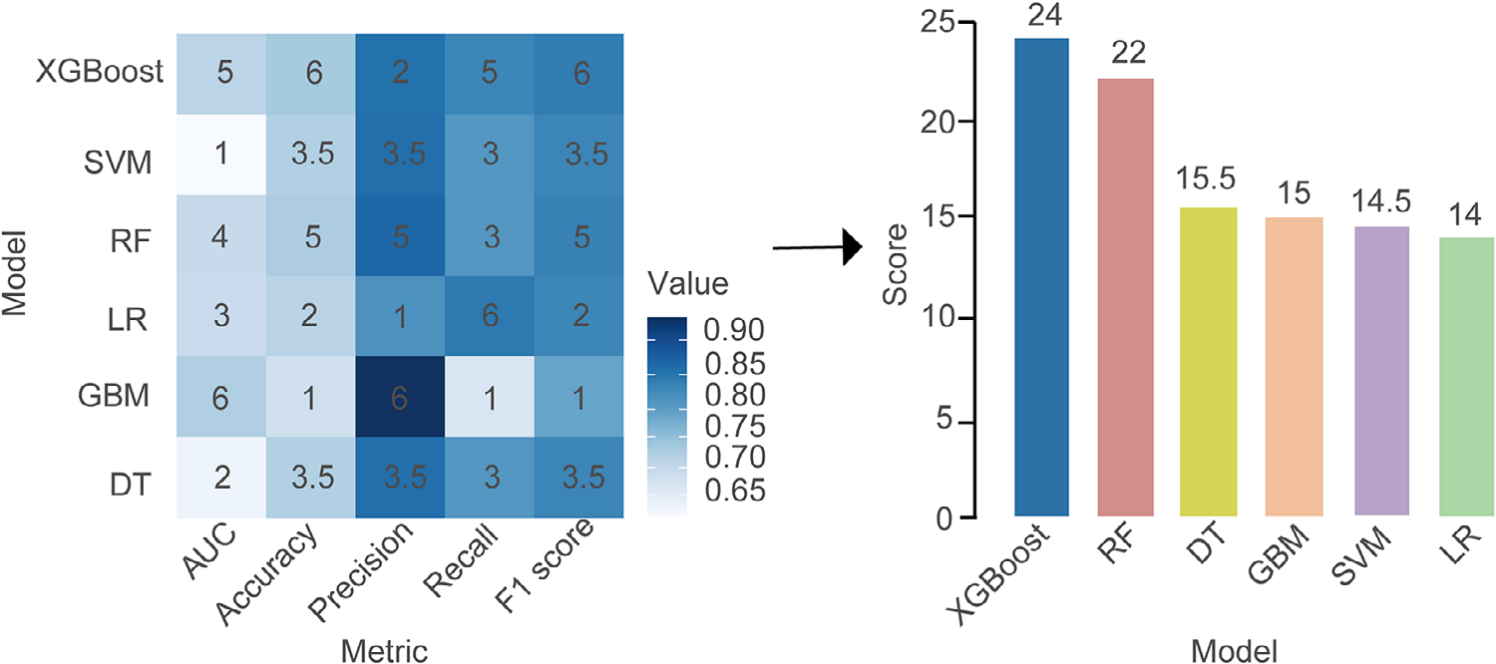
Performance comparison of various ML models. Value represents the size of the five evaluation index values, and the values in the graphic color blocks represent the model ranking under each index. AUC, area under the curve.

We regarded the people with initial values and high overall levels in the anxiety or depression trajectory group as the high‐risk group of negative emotions, namely the “Gradually increasing high‐anxiety group” and the “High‐progressive depression group”, a total of 157 people, and the remaining 703 people were non‐high‐risk groups. In the model evaluation, the test data set is used to select the area under the curve (AUC), accuracy, precision, recall, and F1 score of the discrimination index to evaluate the prediction performance of the improved model. The results of all evaluation indicators are summarized in Table 4. Figure 4 shows the heat map of predictive performance data visualization in all models. The results show that the prediction performance score of extreme gradient boosting (XGBoost) ranks first (24 points), followed by random forest (RF) (22 points) and decision tree (DT) (15.5 points). These results show that the XGBoost algorithm performs best in developing predictive models to estimate high‐risk groups of perinatal negative emotions.

**TABLE 4:** Performance fitting index of ML model.

Model	AUC	Accuracy	Precision	Recall	F1 score
XGBoost	0.72	0.74	0.85	0.83	0.84
RF	0.71	0.74	0.86	0.81	0.84
DT	0.66	0.73	0.86	0.81	0.83
GBM	0.73	0.70	0.93	0.69	0.79
SVM	0.65	0.73	0.86	0.81	0.83
LR	0.71	0.73	0.82	0.85	0.83

Abbreviations: XGBoost: extreme gradient boosting, RF: random forest, DT: decision tree, GBM: gradient boosting machine, SVM: support vector machine, LR: logistic regression, AUC: area under the curve.

### 2.6 Model Interpretability and Feature Importance

Based on the XGBoost model, the variables are sorted by model interpretability and their contributions to the outcome are visualized (Figures S1 and S2). Among the 15 indicator variables, whether to receive school education and social support for pregnant women has the greatest impact on trajectory classification, and its impact is significantly higher than that of other indicators. In addition to these two indicators, the variables that affect the decreasing importance of high risk of negative emotions are as follows: receiving one to five times of pregnant women school education, moderate stress, passive smoking, more than 15 times of prenatal examination, high level of social support, on‐the‐job status, mild stress, 6–15 times of prenatal examination, good family function, moderate family dysfunction, etc. In the XGBoost algorithm, the relative contribution of features to the algorithm is positively or negatively correlated with high‐risk groups (Figure S1A). This paper also provides a typical example to illustrate the interpretability of the model. A perinatal woman in a non‐high‐risk group had a lower SHAP value prediction score (−0.42) (Figure S1B).

## 3 Discussion

This study explores the developmental trajectory of negative emotions among Chinese perinatal women from a dynamic perspective. It innovatively applies multiple ML models to analyze dynamic data simultaneously and utilizes SHAP to interpret the optimal model. This approach aims to construct a high‐risk identification model for negative emotions throughout the perinatal period, providing valuable insights for clinical medical staff.

In this study, the detection rates of anxiety symptoms were 13.95%, 11.63%, 10.12%, and 11.63%, respectively, while the detection rates of depression symptoms were 5.12%, 4.53%, 3.37%, and 8.84%, respectively. Utilizing longitudinal data from the follow‐up study of the pregnancy cohort, we identified four trajectories of negative emotions among perinatal women in western China. In terms of anxiety symptoms, we identified two trajectory groups, whereas Huizink et al. [28] and Bayrampour et al. [29] identified five trajectory groups, and Kuo et al. [30] summarized four trajectory groups of perinatal anxiety. In this study, the low‐risk anxiety group had mild symptoms in early pregnancy, which was consistent with previous studies [29]. In terms of depressive symptoms, the “Mild sustained depression group” included 89.4% of women, which was consistent with the results of Mora et al. and Sutter‐Dallay et al. [31, 32], while only 50% of women such as Ahmed met this feature, or due to different depression screening tools and sample characteristics [33]. This shows that the negative emotional symptoms of perinatal women change dynamically with time and are not constant, suggesting that attention should be paid to their dynamic development trend.

Among the 860 perinatal women, 84.40% had anxiety, and 89.40% had a lower risk of depression. In general, the level of anxiety or depression in most women is relatively stable, but the level of anxiety or depression in the “gradually increasing high‐anxiety group” and the “high‐progressive depression group” is higher in the middle and late pregnancy, and decreases after delivery, which is consistent with the conclusion of Asma et al. [33]. It is speculated that the physiological and psychological pressure during pregnancy accumulates with the progress of pregnancy and reaches the peak in the middle and late stages, which may increase the risk of fetal neurodevelopmental abnormalities [11]. Studies have confirmed a correlation between depression during pregnancy and pregnancy‐related stress and anxiety [34, 35]. Therefore, it is essential to strengthen attention to the mental health of prenatal women and to prevent the exacerbation of negative emotions, which could lead to adverse outcomes such as postpartum depression.

Perinatal negative emotion is an important cause of pregnancy complications. Early identification is of great significance to maternal and child health management. In view of its high concealment and prolonged course of disease, timely screening of high‐risk groups is particularly critical. In this study, the synthetic minority oversampling technique (SMOTE) algorithm is used to deal with category imbalance in the modeling process, and the recognition ability of the model to high‐risk individuals is improved by synthesizing minority samples, which ensures the balance of training data. It should be pointed out that although SMOTE can effectively alleviate the problem of data imbalance, it may also lead to overfitting, and the generalization ability of the model still needs to be carefully evaluated. Drawing on Garbazza et al.’s [36] application experience in a large‐scale maternal database, we systematically compared the performance of six classical ML algorithms, RF, logistic regression (LR), support vector machine (SVM), XGBoost, gradient boosting machine (GBM), and DT, in predicting perinatal negative emotions using multicenter longitudinal data. The results show that the XGBoost model has the best prediction ability and is superior to other models such as RF and LR. In contrast, traditional LR is limited by linear assumptions and performs poorly when dealing with complex nonlinear feature interactions, while XGBoost can efficiently process missing data. With the advantages of ensemble learning, it corrects misclassification by constructing a classifier ensemble to improve model accuracy and performance [37, 38]. At the same time, the superiority of the XGBoost model has also been verified in other studies. For example, Zhao et al. [39] reported AUC values of 0.755 (95% CI: 0.719–0.793) and 0.719 (95% CI: 0.675–0.761) in the prediction of depression risk in chronic obstructive pulmonary disease, respectively, which were significantly better than other ML algorithms. Hochman et al. [40] also found that it was stable in the prediction of postpartum depression, with an AUC value of 0.712 (95% CI: 0.690–0.733). These results support the broad applicability and good migration ability of the XGBoost model in the field of mental health.

The interpretability of the XGBoost model was further analyzed, and the SHAP method quantified the contribution of each predictive feature. It was found that the number of school education, social support level, stress, past medical history, smoking, and the number of prenatal examinations were the main contributors. Among these characteristics, the number of school education for pregnant women and the level of social support are considered to be the most important indicators. Relevant studies have shown that maternal school education and adaptation to physiological changes during pregnancy may contribute to the psychological self‐regulation of pregnant women [29, 41]. At the same time, Hemmatipour et al. [42] found that there is a correlation between the level of education during pregnancy and the overall health status. Individuals with higher levels of education tend to be able to better understand health knowledge and adopt more scientific coping styles, thereby improving their overall health status. It is worth noting that social support is also a factor closely related to the occurrence of perinatal negative emotions. The positive effect of social support on emotional health has been confirmed in many studies [29, 34]. During pregnancy, women need more social support than ever before to help them cope with the negative emotions and stress associated with pregnancy. Family support, friend support, and professional support of the medical system can significantly enhance the psychological resilience of pregnant women and prepare them for subsequent pregnancy and childbirth [43]. This suggests that strengthening the construction of health education and social support networks during pregnancy is of practical significance for emotional intervention. Stress levels in this study also showed that there was a higher risk of anxiety symptoms during pregnancy with moderate stress than without stress (OR = 3.29, 95% CI: 1.27–8.52), re‐emphasizing the importance of stress assessment and coping strategies during pregnancy. Past medical history [44] and unhealthy lifestyles such as smoking should also be the focus of attention [45, 46]. In addition, increasing the number of prenatal examinations not only contributes to physical health monitoring but may also be an effective window for screening and intervention for psychological problems.

The samples of this study are mainly from the southwest of China, covering the Han nationality and many ethnic minorities, with strong representativeness and diversity. As a regional medical center, Chongqing attracts a large number of medical patients in the surrounding areas, providing a wider population base for the sample. However, there are still several limitations worth considering. To begin with, the sample of the study is limited to Southwest China, which may limit the promotion of the research results to other regions. Therefore, when applying the research results to other regions, the population differences in the region should be carefully considered. Second, there are potential biases in the self‐reported questionnaire data, such as social expectation bias and memory bias, which may affect the accuracy of the data. In addition, the structural limitations of the questionnaire also lead to the possible omission of some important influencing factors. Future research can be improved by optimizing the questionnaire design or combining other data sources.

## 4 Materials and Methods

### 4.1 Study Design and Participants

This study is observational, and the data come from the “Chongqing Maternal Cohort” established by the team. The cohort received funding from the National Natural Science Foundation of China (project number: 71573027) and was approved by the Ethics Committee of Chongqing Medical University. It is a longitudinal follow‐up study conducted in Chongqing, a provincial city in southwestern China, from March 1, 2018, to January 31, 2019. Using a structured questionnaire, we performed a longitudinal follow‐up survey of perinatal women at four large hospitals. The survey time points included the first trimester (less than 15 weeks), the second trimester (15–27 weeks), the third trimester (28 weeks or more), and approximately 6 weeks postpartum. We collected information on their mental health status, which encompassed demographic data, personal lifestyle factors, prenatal records, family dynamics, social influences, and other multidimensional information. A total of 860 perinatal women with complete demographic data, personal lifestyle, prenatal records, family factors, social factors, and other information were included in the study. Baseline data were obtained at the time of enrollment, and these were utilized to assess the perinatal trajectory of anxiety and depression symptoms.

### 4.2 Measurement of Negative Emotional Symptoms

This study focused on the negative emotions experienced by perinatal women, specifically anxiety and depression, and evaluated the participants using standardized scales. The Hamilton Anxiety Scale (HAMA) was employed to measure anxiety [47]. The scale demonstrated good reliability. The Cronbach's α coefficients for early pregnancy, middle pregnancy, late pregnancy, and postpartum were 0.891, 0.914, 0.918, and 0.930, respectively, all exceeding 0.8. The assessment process was conducted collaboratively by two trained raters who scored independently. According to the HAMA scores, severe anxiety was defined as a total score exceeding 30 points, moderate anxiety as a score between 22 and 29 points, and scores below 15 points were classified as the nonanxiety group.

Depressive symptoms were evaluated using the self‐rating depression scale (SDS) [48]. This scale consists of 20 items, each representing a specific symptom. A total score of less than 0.5 indicates no depression, a score between 0.50 and 0.59 indicates mild depression, a score between 0.60 and 0.69 indicates moderate depression, and a score of 0.70 or higher indicates severe depression. The Cronbach's α coefficients for the SDS during early pregnancy, middle pregnancy, late pregnancy, and postpartum were 0.794, 0.820, 0.827, and 0.887, respectively.

### 4.3 Growth Mixture Model

GMM assumes that the data consists of a mixture of several Gaussian distributions [49]. Each Gaussian distribution represents a potential category, and each data point is generated from these Gaussian distributions with a specific probability. By estimating the parameters of these Gaussian distributions, we can describe the overall distribution of the data and infer the potential categories to which the data points belong. Determining the number of potential categories is crucial for fitting GMM, especially when significant differences exist in emotional trajectories among individuals. Traditional models typically assume that all individuals share a single trajectory, which overlooks individual variability. GMM can more accurately capture the diverse emotional patterns within the perinatal population by identifying potential subgroups. We identify the best‐fitting model and the most appropriate latent class based on information criteria and model fit test results. Commonly used information criteria include AIC, BIC, aBIC, and Entropy, as well as LMR and BLRT. We select the optimal category model based on higher entropy values and lower AIC, BIC, and aBIC values. Based on higher entropy and lower AIC, BIC, and aBIC values, the optimal categorical model is selected. For the classification model, the *p‐*values of the LMR and BLRT tests are significantly reduced, indicating that these models outperform others. It is generally accepted that values greater than 0.80 indicate well‐separated clusters.

### 4.4 ML Model

ML‐based clinical prediction models have demonstrated significant advantages in the early screening and risk assessment of clinical diseases. These models can enhance the accuracy and efficiency of disease risk identification through data‐driven modeling methods [50]. RF algorithm is a widely used ML technique that employs a self‐sampling method. It is particularly effective for handling missing values in high‐dimensional data [51]. In the context of model development, the complete dataset is first divided into a training set and a test set in an 8:2 ratio. To address the common issue of class imbalance in perinatal psychological data, the SMOTE is employed to balance the training set samples [52]. This technique enhances the representativeness of underrepresented categories by generating synthetic samples. During the model construction phase, statistical analysis and domain knowledge are integrated to establish a feature set based on the results of univariate analysis and relevant risk factors identified in previous literature [18, 28, 53]. Six classical ML algorithms are utilized to develop prediction models, including LR, RF, SVM, XGBoost, GBM, and DT.

Each algorithm performs hyperparameter optimization using grid search in conjunction with 10‐fold cross‐validation. This process is repeated 10 times to mitigate the effects of randomness, and ultimately, the parameter configuration that yields the best average performance on the validation set is selected. Model validation employs a multidimensional evaluation system to compute key metrics, including AUC, accuracy, precision, recall, and F1 score on the independent test set. In accordance with the validation criteria established in previous prediction models [54, 55], a quantitative scoring mechanism is implemented: each evaluation metric is ranked based on model performance, with the highest performance receiving 6 points and the lowest receiving 1 point. Ultimately, the optimal model is determined through a comprehensive scoring method, whereby the model with the highest cumulative score across all metrics is selected as the final prediction tool for identifying high‐risk groups for perinatal negative emotions.

### 4.5 Statistical Analysis

According to the research content, SPSS 23 was used to test the reliability of the scales involved. GMM was established by the Mplus7.0 software to estimate the average growth curve and growth factor variance of each subgroup, to explore the dynamic developmental trajectory of perinatal anxiety and depression. Chi‐square analysis and multiple LR models were performed using SAS 9.4 to explore the significant correlation between maternal characteristics and different trajectory subgroups. Finally, R 4.2.3 was used to construct an ML prediction model to provide support for early identification of high‐risk pregnant women with negative emotions during the perinatal period.

## 5 Conclusion

This study revealed four dynamic developmental trajectories of anxiety and depression during the perinatal period: a low‐stable anxiety group, a gradually increasing high‐anxiety group, a mild sustained depression group, and a high‐progressive depression group. It also identified predictive factors influencing these emotional trajectories, underscoring the importance of managing perinatal mental health. Consequently, it is advisable to integrate mental health assessments into the routine nursing care for pregnant women and to increase the frequency of psychological evaluations. This approach aims to ensure comprehensive and individualized mental health support for pregnant women, thereby enhancing the capacity for early detection and effective intervention in the development of negative emotional trajectories. In addition, the negative emotional risk prediction model based on the XGBoost algorithm demonstrates strong performance, providing a feasible and effective tool for the early identification of high‐risk pregnant women and assisting in the development of targeted prevention and intervention measures for this population.

## Author Contributions

Conceptualization: Yuan Zhang and Wenlong Li; methodology: Jian Zou; software: Wenlong Li; validation: Guohui Yang; formal analysis: Yuan Zhang; investigation: Wenlong Li; resources: Jian Zou; data curation: Xiaoni Zhong; writing – original draft preparation: Yuan Zhang; writing‐review and editing: Yuan Zhang; visualization: Wenlong Li; supervision: Jian Zou; project administration: Biao Xie; funding acquisition: Biao Xie. All authors have read and agreed to the published version of the manuscript.

## Conflicts of Interest

The authors declare no conflicts of interest.

## Ethics Statement

The study protocol was approved by the Ethics Committee of Chongqing Medical University (approval time: 2015.11.30). All participants signed the informed consent.

## Supporting information




**Table S1**. The occurrence of perinatal anxiety and depression.
**Table S2**. The classification fitting information of each latent class by the GMM (anxiety).
**Table S3**. The classification fitting information of each latent class by the GMM (depression).
**Table S4**. Results of single‐factor analysis of anxiety trajectory.
**Table S5**. Results of single‐factor analysis of depression trajectory.
**Figure S1**. SHAP of the model. (**A**) The importance ranking diagram of XGBoost model features and the feature attributes in SHAP. (**B**) No. 85 sample waterfall diagram.
**Figure S2**. The partial correlation dependence graph of the top six variables.

## Data Availability

The datasets used and/or analyzed during the current study are available from the corresponding author on reasonable request.
