# Supplementary material for: Using Machine Learning to Identify the Dynamic Evolution Patterns of Negative Emotions in Perinatal Women: A Longitudinal Study in Southwest China
Source: MedComm (2020). 2025 Aug 15;6(8):e70331. doi: 10.1002/mco2.70331 (PMC12356827; doi:10.1002/mco2.70331)
Supplement: Supplementary file 1 — Table S1. The occurrence of perinatal anxiety and depression. Table S2. The classification fitting information of each latent class by the GMM (anxiety). Table S3. The classification fitting information of each latent class by the GMM (depression). Table S4. Results of single‐factor analysis of anxiety trajectory. Table S5. Results of single‐factor analysis of depression trajectory. Figure S1. SHAP of the model. (A) The importance ranking diagram of XGBoost model features and the feature attributes in SHAP. (B) No. 85 sample waterfall diagram. Figure S2. The partial correlation dependence graph of the top six variables. [file MCO2-6-e70331-s001.docx]

**Using machine learning to identify the dynamic evolution patterns of negative emotions in perinatal women: A longitudinal study in Southwest China**

Yuan Zhang ^1,2,^ ^#^, Wenlong Li ^1,2,^ ^#^, Jian Zou ^1,2,^ ^#^, Guohui Yang ^1,2^, Xiaoni Zhong ^1,2^, Biao Xie ^1,2, *^

^1^Department of Epidemiology and Health Statistics, School of Public Health, Chongqing Medical University, Yixue Road, Chongqing 400016, China

^2^Research Center for Medicine and Social Development, Chongqing Medical University, Chongqing, 400016, China

^#^These authors contribute equally

^*^Corresponding author: kybiao@cqmu.edu.cn (B.X.)

**Table S1**. The occurrence of perinatal anxiety and depression.

| Period | Anxiety (%) | Depression (%) |
| --- | --- | --- |
| Early pregnancy |  |  |
| No | 86.05 | 94.88 |
| Mild | 10.81 | 4.65 |
| Moderate | 2.44 | 0.23 |
| Severe | 0.70 | 0.23 |
| Mid pregnancy |  |  |
| No | 88.37 | 95.47 |
| Mild | 7.44 | 3.60 |
| Moderate | 3.72 | 0.81 |
| Severe | 0.47 | 0.12 |
| Late pregnancy |  |  |
| No | 89.88 | 96.63 |
| Mild | 7.91 | 3.02 |
| Moderate | 2.09 | 0.35 |
| Severe | 0.12 | 0.00 |
| Postpartum |  |  |
| No | 88.37 | 91.16 |
| Mild | 7.56 | 6.63 |
| Moderate | 3.49 | 1.86 |
| Severe | 0.58 | 0.35 |

**Table S2**. The classification fitting information of each latent class by the GMM (anxiety).

|  | Entropy | LMR | BLRT | Class probability |
| --- | --- | --- | --- | --- |
| Linear estimation |  |  |  |  |
| CLASS-1 |  |  |  |  |
| CLASS-2 | 0.834 | 0.020 | 0.000 | 0.85698/0.14302 |
| CLASS-3 | 0.874 | 0.022 | 0.000 | 0.01860/0.78140/0.20000 |
| CLASS-4 | 0.866 | 0.029 | 0.000 | 0.05/0.01977/0.17558/0.75465 |
| CLASS-5 | 0.849 | 0.572 | 0.000 | 0.16860/0.72558/0.04186/0.01512/0.04884 |
| Quadratic estimation |  |  |  |  |
| CLASS-1 |  |  |  |  |
| CLASS-2 | 0.863 | 0.006 | 0.000 | 0.84419/0.15581 |
| CLASS-3 | 0.853 | 0.205 | 0.000 | 0.05581/0.13488/0.80930 |
| CLASS-4 | 0.846 | 0.311 | 0.000 | 0.05581/0.11628/0.76512/0.06279 |
| CLASS-5 | 0.867 | 0.369 | 0.000 | 0.71860/0.05349/0.04535/0.15930/0.02326 |
| Free estimation |  |  |  |  |
| CLASS-1 |  |  |  |  |
| CLASS-2 | 0.854 | 0.009 | 0.000 | 0.88837/0.11163 |
| CLASS-3 | 0.875 | 0.000 | 0.000 | 0.04419/0.25116/0.70465 |
| CLASS-4 | 0.918 | 0.427 | 0.000 | 0.04419/0.03488/0.08721/0.83372 |
| CLASS-5 | 0.915 | 0.135 | 0.000 | 0.01279/0.03953/0.07209/0.09535/0.78023 |

CLASS-1-CLASS-5 represents the model when the population is divided into 1-5 categories. Abbreviations: LMR, Lo-mendell-rubin; BLRT, Bootstrapped likelihood ratio test.

**Table S3.** The classification fitting information of each latent class by the GMM (depression).

| Model | Entropy | LMR | BLRT | Class probability |
| --- | --- | --- | --- | --- |
| Linear estimation |  |  |  |  |
| CLASS-1 |  |  |  |  |
| CLASS-2 | 0.937 | 0.026 | 0.000 | 0.90233/0.09767 |
| CLASS-3 | 0.942 | 0.0844 | 0.000 | 0.0186/0.10465/0.87674 |
| CLASS-4 | 0.891 | 0.2435 | 0.000 | 0.76744/0.01860/0.13140/0.08256 |
| CLASS-5 | 0.918 | 0.0112 | 0.000 | 0.02558/0.07326/0.00233/0.14302/0.75581 |
| Quadratic estimation |  |  |  |  |
| CLASS-1 |  |  |  |  |
| CLASS-2 | 0.943 | 0.0035 | 0.000 | 0.89535/0.10465 |
| CLASS-3 | 0.920 | 0.1036 | 0.000 | 0.83023/0.11279/0.05698 |
| CLASS-4 | 0.919 | 0.3575 | 0.000 | 0.07326/0.79419/0.01977/0.11279 |
| CLASS-5 | 0.922 | 0.5739 | 0.000 | 0.76628/0.02326/0.01744/0.10233/0.09070 |
| Free estimation |  |  |  |  |
| CLASS-1 |  |  |  |  |
| CLASS-2 | 0.934 | 0.0052 | 0.000 | 0.88023/0.11977 |
| CLASS-3 | 0.922 | 0.0288 | 0.000 | 0.04651/0.15465/0.79884 |
| CLASS-4 | 0.931 | 0.1059 | 0.000 | 0.78837/0.15465/0.04535/0.01163 |
| CLASS-5 | 0.910 | 0.2335 | 0.000 | 0.06395/0.03721/0.76163/0.11047/0.02674 |

**Table S4.** Results of single factor analysis of anxiety trajectory.

| Factors | Total | Non-high-risk | High-risk | Statistic | | *p* |
| --- | --- | --- | --- | --- | --- | --- |
|  | *n* (%) | *n* (%) | *n* (%) |  |  |  |
|  | 860 (100) | 726 (84.42) | 134 (15.58) |  | |  |
| Ethnic groups |  |  |  | $\chi^{2}$=0.00 | | 1.00 |
| Han nationality | 840 (97.67) | 709 (97.66) | 131 (97.76) |  | |  |
| Other nationalities | 20 (2.33) | 17 (2.34) | 3 (2.24) |  | |  |
| Place of residence |  |  |  | $\chi^{2}$=1.30 | | 0.25 |
| Towns | 532 (61.86) | 455 (62.67) | 77 (57.46) |  | |  |
| Rural | 328 (38.14) | 271 (37.33) | 57 (42.54) |  | |  |
| Educational level |  |  |  | H=0.31 | | 0.86 |
| Junior high school and below | 278 (32.33) | 232 (31.96) | 46 (34.33) |  | |  |
| High school/vocational high school/technical secondary school | 254 (29.54) | 215 (29.61) | 39 (29.10) |  | |  |
| Junior college/undergraduate and above | 328 (38.14) | 279 (38.43) | 49 (36.57) |  | |  |
| Profession |  |  |  | $\chi^{2}$=4.14 | 0.042* | |
| On the job | 416 (48.37) | 362 (49.86) | 54 (40.30) |  |  | |
| Housewives/unemployed | 444 (51.63) | 364 (50.14) | 80 (59.70) |  |  | |
| Family per capita monthly income |  |  |  | $\chi^{2}$=5.36 | 0.07 | |
| ≤3000 | 223 (25.93) | 188 (25.90) | 35 (26.12) |  |  | |
| 3001-5000 | 357 (41.51) | 291 (40.08) | 66 (49.25) |  |  | |
| ≥5001 | 280 (32.56) | 247 (34.02) | 33 (24.63) |  |  | |
| Medical expenses payment method |  |  |  | $\chi^{2}$=0.24 | 0.62 | |
| Private expense | 343 (39.88) | 287 (39.53) | 56 (41.79) |  |  | |
| Medical insurance | 517 (60.12) | 439 (60.47) | 78 (58.21) |  |  | |
| Exercise during pregnancy |  |  |  | $\chi^{2}$=0.21 | 0.65 | |
| Yes | 453 (52.67) | 380 (52.34) | 73 (54.48) |  |  | |
| No | 407 (47.33) | 346 (47.66) | 61 (45.52) |  |  | |
| Accept pregnant women school education |  |  |  | $\chi^{2}$=2.21 | 0.14 | |
| Yes | 102 (11.86) | 81 (11.16) | 21 (15.67) |  |  | |
| No | 758 (88.14) | 645 (88.84) | 113 (84.33) |  |  | |
| Number of times of receiving pregnancy education |  |  |  | $\chi^{2}$=7.21 | 0.027* | |
| 0 | 747 (86.86) | 640 (88.15) | 107 (79.85) |  |  | |
| 1-5 | 97 (11.28) | 73 (10.06) | 24 (17.91) |  |  | |
| ≥6 | 16 (1.86) | 13 (1.79) | 3 (2.24) |  |  | |
| Degree of care of doctors and nurses |  |  |  | $\chi^{2}$=3.74 | 0.05 | |
| Good | 803 (93.37) | 683 (94.08) | 120 (89.55) |  |  | |
| Bad | 57 (6.63) | 43 (5.92) | 14 (10.45) |  |  | |

| Factors | Total | Non-high-risk | High-risk | Statistic | *p* |
| --- | --- | --- | --- | --- | --- |
|  | *n* (%) | *n* (%) | *n* (%) |  |  |
| Surrounding maternal mode of delivery |  |  |  | $\chi^{2}$=0.38 | 0.83 |
| More vaginal births | 345 (40.12) | 292 (40.22) | 53 (39.55) |  |  |
| There are more cesarean section | 128 (14.88) | 110 (15.15) | 18 (13.43) |  |  |
| The two are almost the same | 387 (45.00) | 324 (44.63) | 63 (47.02) |  |  |
| I intend the mode of delivery |  |  |  | $\chi^{2}$=4.42 | 0.11 |
| Uncertain | 371 (43.14) | 321 (44.22) | 50 (37.31) |  |  |
| Vaginal delivery | 446 (51.86) | 366 (50.41) | 80 (59.70) |  |  |
| Cesarean section | 43 (5.00) | 39 (5.37) | 4 (2.99) |  |  |
| Age |  |  |  | H=1.04 | 0.56 |
| <25 | 374 (43.49) | 311 (42.84) | 63 (47.02) |  |  |
| 25-30 | 365 (42.44) | 310 (42.70) | 55 (41.05) |  |  |
| >30 | 121 (14.07) | 105 (14.46) | 16 (11.94) |  |  |
| history of drinking |  |  |  | $\chi^{2}$=0.68 | 0.41 |
| Yes | 10 (1.16) | 7 (0.96) | 3 (2.24) |  |  |
| No | 850 (98.84) | 719 (99.04) | 131 (97.76) |  |  |
| Smoking history |  |  |  | $\chi^{2}$=3.59 | 0.06 |
| Active | 26 (3.02) | 18 (2.48) | 8 (5.97) |  |  |
| Passive | 834 (96.98) | 708 (97.52) | 126 (94.03) |  |  |
| Previous medical history |  |  |  | $\chi^{2}$=6.81 | 0.009** |
| Yes | 58 (6.74) | 42 (5.79) | 16 (11.94) |  |  |
| No | 802 (93.26) | 684 (94.22) | 118 (88.06) |  |  |
| History of pregnancy |  |  |  | $\chi^{2}$=3.31 | 0.07 |
| Yes | 618 (71.86) | 513 (70.66) | 105 (78.36) |  |  |
| No | 242 (28.14) | 213 (29.34) | 29 (21.64) |  |  |
| BMI |  |  |  | H=0.22 | 0.90 |
| <18.5 | 140 (16.28) | 120 (16.53) | 20 (14.93) |  |  |
| 18.5-25 | 649 (75.47) | 546 (75.21) | 103 (76.87) |  |  |
| >25 | 71 (8.26) | 60 (8.26) | 11 (8.21) |  |  |
| Knowledge level of childbirth |  |  |  | H=2.75 | 0.25 |
| Low level | 350 (40.70) | 303 (41.74) | 47 (35.08) |  |  |
| Medium level | 425 (49.42) | 350 (48.21) | 75 (55.97) |  |  |
| High level | 85 (9.88) | 73 (10.06) | 12 (8.96) |  |  |
| Family care degree |  |  |  | H=5.22 | 0.07 |
| Serious dysfunction of family function | 55 (6.40) | 41 (5.65) | 14 (10.45) |  |  |
| Moderate family dysfunction | 226 (26.28) | 188 (25.90) | 38 (28.36) |  |  |
| The family function is good | 579 (67.33) | 497 (68.46) | 82 (61.20) |  |  |
| Pregnancy pressure |  |  |  | H=23.45 | <0.001*** |
| No pressure | 63 (7.33) | 57 (7.85) | 6 (4.48) |  |  |
| Mild pressure | 663 (77.09) | 574 (79.06) | 89 (66.42) |  |  |
| Moderate pressure | 128 (14.88) | 90 (12.40) | 38 (28.36) |  |  |
| Severe pressure | 6 (0.70) | 5 (0.69) | 1 (0.75) |  |  |
| Social support |  |  |  | H=24.59 | <0.001*** |
| Low level | 181 (21.05) | 132 (18.18) | 49 (36.57) |  |  |
| Medium level | 545 (63.37) | 472 (65.01) | 73 (54.48) |  |  |
| High level | 134 (15.58) | 122 (16.80) | 12 (8.96) |  |  |
| Number of prenatal examinations |  |  |  | H=4.49 | 0.11 |
| <6 times | 73 (8.49) | 66 (9.09) | 7 (5.22) |  |  |
| 6-15 times | 661 (76.86) | 560 (77.14) | 101 (75.37) |  |  |
| >15 times | 126 (14.65) | 100 (13.77) | 26 (19.40) |  |  |
| Domestic pets |  |  |  | $\chi^{2}$=0.36 | 0.55 |
| Yes | 28 (3.26) | 22 (3.03) | 6 (4.48) |  |  |
| No | 832 (96.74) | 704 (96.97) | 128 (95.52) |  |  |

$\chi^{2}$: Chi-square test; H: Kruskal-Wallis H Test. **p* < 0.05.***p* < 0.01. ****p* < 0.001.

**Table S5.** Results of single factor analysis of depression trajectory.

| Factors | Total | Non-high-risk | High-risk | Statistic | *p* |
| --- | --- | --- | --- | --- | --- |
|  | *n* (%) | *n* (%) | *n* (%) |  |  |
|  | 860 (100) | 770 (89.54) | 90 (10.46) |  |  |
| Ethnic groups |  |  |  | $\chi^{2}$=0.19 | 0.66 |
| Han nationality | 840 (97.7) | 751 (97.53) | 89 (98.89) |  |  |
| Other nationalities | 20 (2.33) | 19 (2.47) | 1 (1.11) |  |  |
| Place of residence |  |  |  | $\chi^{2}$=1.15 | 0.29 |
| Towns | 532 (61.86) | 481 (62.47) | 51 (56.67) |  |  |
| Rural | 328 (38.14) | 289 (37.53) | 39 (43.33) |  |  |
| Educational level |  |  |  | H=2.15 | 0.34 |
| Junior high school and below | 278 (32.33) | 249 (32.34) | 29 (32.22) |  |  |
| High school/vocational high school/technical secondary school | 254 (29.54) | 222 (28.83) | 32 (35.56) |  |  |
| Junior college/undergraduate and above | 328 (38.14) | 299 (38.83) | 29 (32.22) |  |  |
| Profession |  |  |  | $\chi^{2}$=4.52 | 0.034* |
| On the job | 416 (48.37) | 382 (49.61) | 34 (37.78) |  |  |
| Housewives/unemployed | 444 (51.63) | 388 (50.39) | 56 (62.22) |  |  |
| Family per capita monthly income |  |  |  | $\chi^{2}$=2.49 | 0.29 |
| ≤3000 | 223 (25.93) | 199 (25.84) | 24 (26.67) |  |  |
| 3001-5000 | 357 (41.51) | 314 (40.78) | 43 (47.78) |  |  |
| ≥5001 | 280 (32.56) | 257 (33.38) | 23 (25.56) |  |  |
| Medical expenses payment method |  |  |  | $\chi^{2}$=1.93 | 0.17 |
| At his own expense | 343 (39.88) | 301 (39.09) | 42 (46.67) |  |  |
| Medical insurance | 517 (60.12) | 469 (60.91) | 48 (53.33) |  |  |
| Exercise during pregnancy |  |  |  | $\chi^{2}$=0.34 | 0.56 |
| Yes | 453 (52.67) | 403 (52.34) | 50 (55.56) |  |  |
| No | 407 (47.33) | 367 (47.66) | 40 (44.44) |  |  |
| Accept pregnant women school education |  |  |  | $\chi^{2}$=3.37 | 0.07 |
| Yes | 102 (11.86) | 86 (11.17) | 16 (17.78) |  |  |
| No | 758 (88.14) | 684 (88.83) | 74 (82.22) |  |  |
| Number of times of receiving pregnancy education |  |  |  | $\chi^{2}$=11.22 | 0.004** |
| 0 | 747 (86.86) | 676 (87.79) | 71 (78.89) |  |  |
| 1-5 | 97 (11.28) | 78 (10.13) | 19 (21.11) |  |  |
| ≥6 | 16 (1.86) | 16 (2.08) | 0 (0.00) |  |  |
| Degree of care of doctors and nurses |  |  |  | $\chi^{2}$=7.30 | 0.007** |
| Good | 803 (93.37) | 725 (94.16) | 78 (86.67) |  |  |
| Bad | 57 (6.63) | 45 (5.84) | 12 (13.33) |  |  |

| Factors | | Total | | Non-high-risk | | High-risk | | Statistic | | *p* | | | |
| --- | --- | --- | --- | --- | --- | --- | --- | --- | --- | --- | --- | --- | --- |
|  |  | *n* (%) | | *n* (%) | | *n* (%) | |  |  |  |  |  |  |
| Surrounding maternal mode of delivery | |  | |  | |  | | $\chi^{2}$=1.40 | | 0.50 | | |  |
| More vaginal births | | 345 (40.12) | | 314 (40.78) | | 31 (34.44) | |  | |  | | |  |
| There are more cesarean section | | 128 (14.88) | | 114 (14.81) | | 14 (15.56) | |  | |  | | |  |
| The two are almost the same | | 387 (45.00) | | 342 (44.42) | | 45 (50.00) | |  | |  | | |  |
| I intend the mode of delivery | |  | |  | |  | | $\chi^{2}$=0.20 | | 0.91 | | |  |
| Uncertain | | 371 (43.14) | | 334 (43.38) | | 37 (41.11) | |  | |  | | |  |
| Vaginal delivery | | 446 (51.86) | | 398 (51.69) | | 48 (53.33) | |  | |  | | |  |
| Cesarean section | | 43 (5.00) | | 38 (4.94) | | 5 (5.56) | |  | |  | | |  |
| Age | |  | |  | |  | | H=0.91 | | 0.64 | | |  |
| <25 | | 374 (43.49) | | 332 (43.12) | | 42 (46.67) | |  | |  | | |  |
| 25-30 | | 365 (42.44) | | 331 (42.99) | | 34 (37.78) | |  | |  | | |  |
| >30 | | 121 (14.07) | | 107 (13.90) | | 14 (15.56) | |  | |  | | |  |
| history of drinking | |  | |  | |  | | $\chi^{2}$=0.22 | | 0.64 | | |  |
| Yes | | 10 (1.16) | | 8 (1.04) | | 2 (2.22) | |  | |  | | |  |
| No | | 850 (98.84) | | 762 (98.96) | | 88 (97.78) | |  | |  | | |  |
| Smoking history | |  | |  | |  | | $\chi^{2}$=6.05 | | 0.014* | | |  |
| Active | | 26 (3.02) | | 19 (2.47) | | 7 (7.78) | |  | |  | | |  |
| Passive | | 834 (96.98) | | 751 (97.53) | | 83 (92.22) | |  | |  | | |  |
| Previous medical history | |  | |  | |  | | $\chi^{2}$=6.94 | | 0.008** | | |  |
| Yes | | 58 (6.74) | | 46 (5.97) | | 12 (13.33) | |  | |  | | |  |
| No | | 802 (93.26) | | 724 (94.03) | | 78 (86.67) | |  | |  | | |  |
| History of pregnancy | |  | |  | |  | | $\chi^{2}$=1.15 | | 0.28 | | |  |
| Yes | | 618 (71.86) | | 549 (71.30) | | 69 (76.67) | |  | |  | | |  |
| No | | 242 (28.14) | | 221 (28.70) | | 21 (23.33) | |  | |  | | |  |
| BMI | |  | |  | |  | | H=1.04 | | 0.59 | | |  |
| <18.5 | | 140 (16.28) | | 123 (15.98) | | 17 (18.89) | |  | |  | | |  |
| 18.5-25 | | 649 (75.47) | | 585 (75.97) | | 64 (71.11) | |  | |  | | |  |
| >25 | | 71 (8.26) | | 62 (8.05) | | 9 (10.00) | |  | |  | | |  |
| Knowledge level of childbirth | |  | |  | |  | | H=2.17 | | 0.34 | | |  |
| Low level | | 350 (40.70) | | 318 (41.30) | | 32 (35.56) | |  | |  | | |  |
| Medium level | | 425 (49.42) | | 374 (48.57) | | 51 (56.67) | |  | |  | | |  |
| High level | | 85 (9.88) | | 78 (10.13) | | 7 (7.78) | |  | |  | | |  |
| Family care degree | |  | |  | |  | | H=7.80 | | 0.020* | | |  |
| Serious dysfunction of family function | | 55 (6.40) | | 46 (5.97) | | 9 (10.00) | |  | |  | | |  |
| Moderate family dysfunction | | 226 (26.28) | | 194 (25.20) | | 32 (35.56) | |  | |  | | |  |
| The family function is good | | 579 (67.33) | | 530 (68.83) | | 49 (54.44) | |  | | | |  | |
| Pregnancy pressure | |  | |  | |  | | H=19.58 | | <0.001*** | | |  |
| No pressure | | 63 (7.33) | | 60 (7.79) | | 3 (3.33) | |  | |  | | |  |
| Mild pressure | | 663 (77.09) | | 604 (78.44) | | 59 (65.56) | |  | |  | | |  |
| Moderate pressure | | 128 (14.88) | | 101 (13.12) | | 27 (30.00) | |  | |  | | |  |
| Factors | | Total | | Non-high-risk | | High-risk | | Statistic | | *p* | | |  |
|  |  | *n* (%) | | *n* (%) | | *n* (%) | |  |  |  |  |  |  |
| Severe pressure | | 6 (0.70) | | 5 (0.65) | | 1 (1.11) | |  | |  | | |  |
| Social support | |  | |  | |  | | H=34.96 | | <0.001*** | | |  |
| Low level | | 181 (21.05) | | 142 (18.44) | | 39 (43.33) | |  | |  | | |  |
| Medium level | | 545 (63.37) | | 497 (64.55) | | 48 (53.33) | |  | |  | | |  |
| High level | | 134 (15.58) | | 131 (17.01) | | 3 (3.33) | |  | |  | | |  |
| Number of prenatal examinations | |  | |  | |  | | H=8.21 | | 0.016* | | |  |
| <6 times | | 73 (8.49) | | 68 (8.83) | | 5 (5.56) | |  | |  | | |  |
| 6-15 times | | 661 (76.86) | | 598 (77.66) | | 63 (70.00) | |  | |  | | |  |
| >15 times | | 126 (14.65) | | 104 (13.51) | | 22 (24.44) | |  | |  | | |  |
| Domestic pets | |  | |  | |  | | $\chi^{2}$=5.02 | | 0.025* | | |  |
| Yes | | 28 (3.26) | | 21 (2.73) | | 7 (7.78) | |  | |  | | |  |
| No | | 832 (96.74) | | 749 (97.27) | | 83 (92.22) | |  | |  | | |  |

$\chi^{2}$: Chi-square test; H: Kruskal-Wallis H Test. **p*<0.05.***p*<0.01. ****p*<0.001.


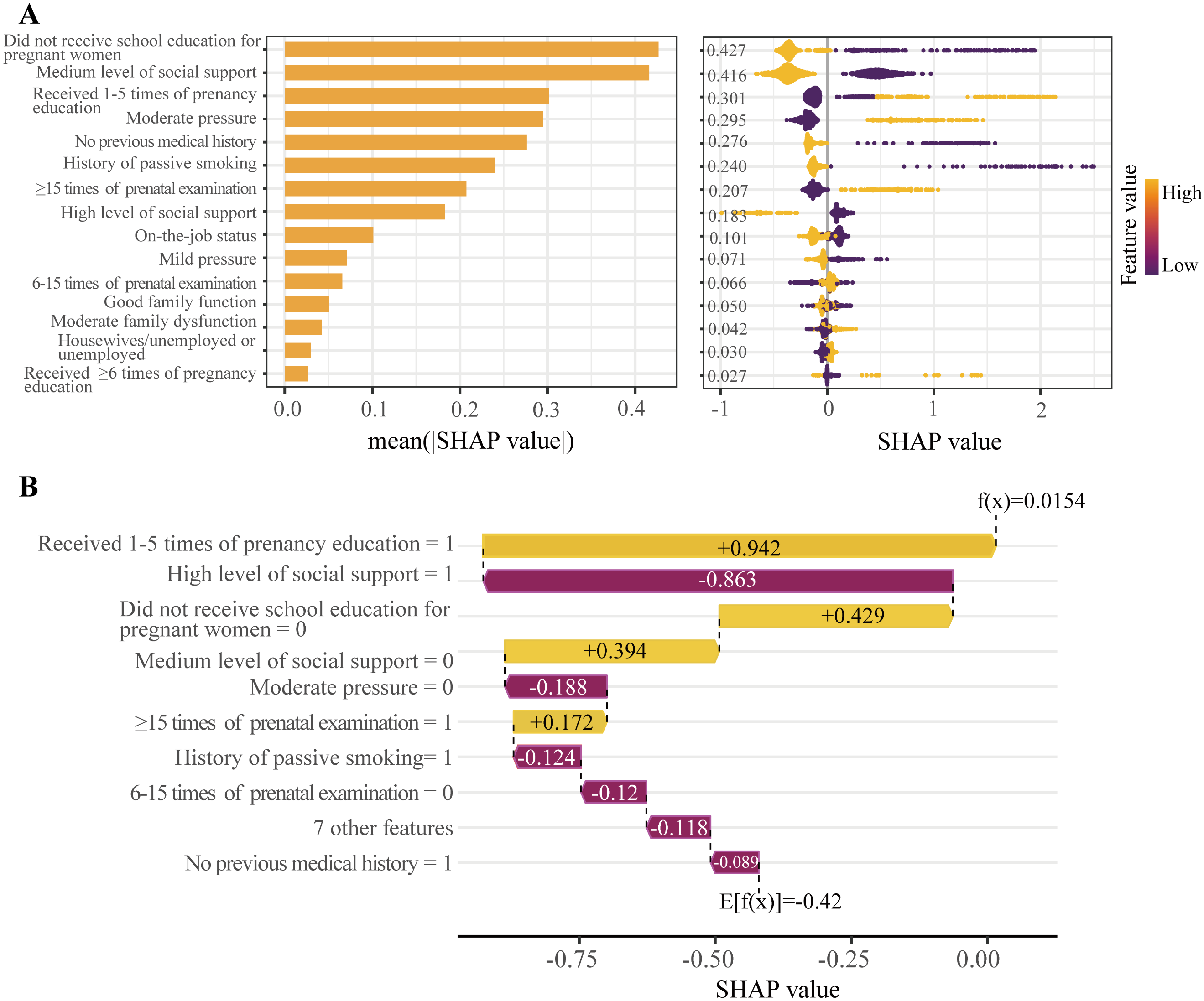


**Figure S1.** SHAP of the model. (**A**) The importance ranking diagram of XGBoost model features and the feature attributes in SHAP. (**B**) No.85 sample waterfall diagram.


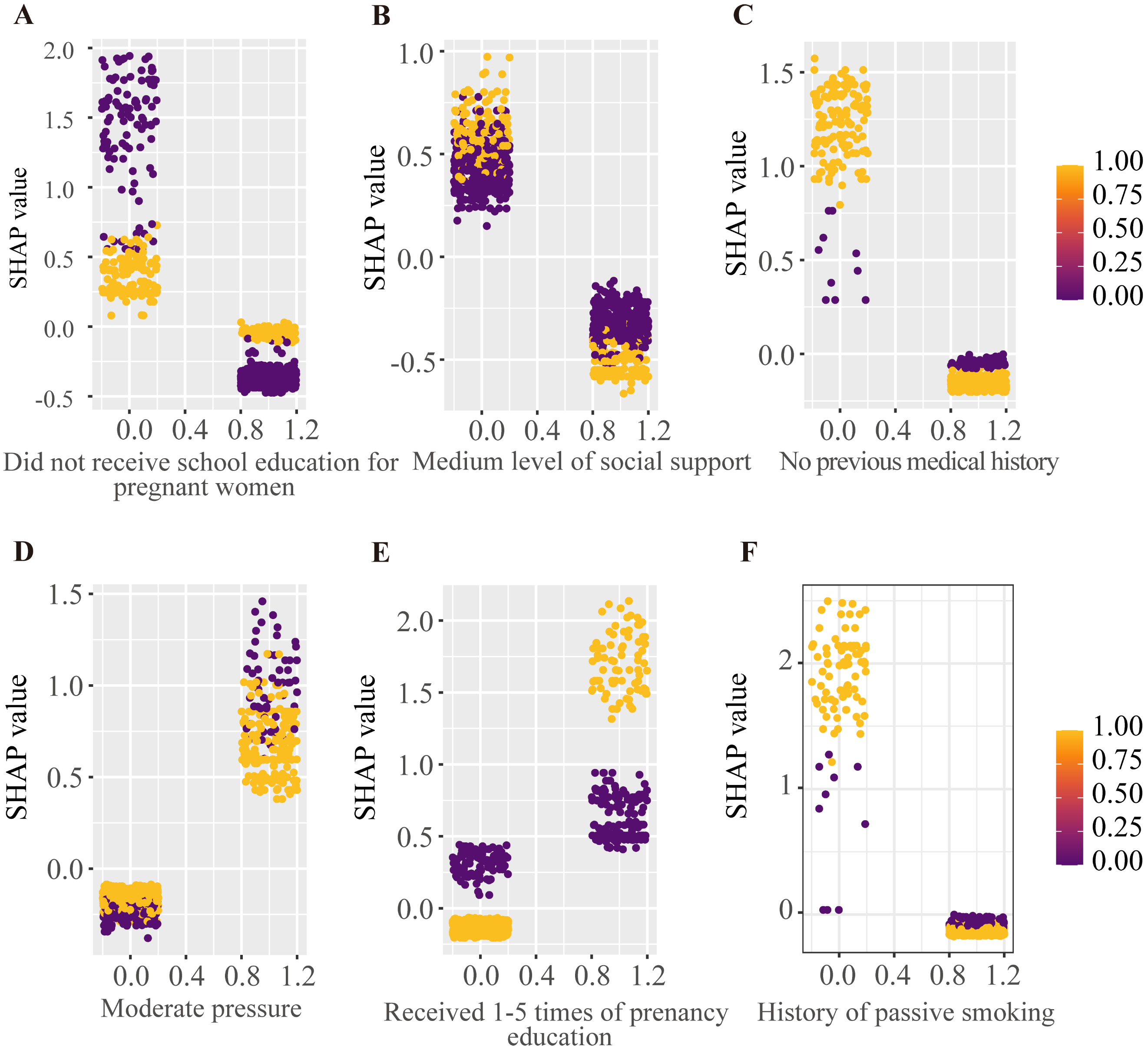


**Figure S2.** The partial correlation dependence graph of the top six variables.
